# Cannabis Use Is Inversely Associated with Metabolic Disorders in Hepatitis C-Infected Patients (ANRS CO22 Hepather Cohort)

**DOI:** 10.3390/jcm11206135

**Published:** 2022-10-18

**Authors:** Tangui Barré, Marc Bourlière, Clémence Ramier, Fabrice Carrat, Vincent Di Beo, Camelia Protopopescu, Fabienne Marcellin, Morgane Bureau, Carole Cagnot, Céline Dorival, Fabien Zoulim, Jessica Zucman-Rossi, Jean-Charles Duclos-Vallée, Hélène Fontaine, Patrizia Carrieri, the ANRS/AFEF Hepather Study Group

**Affiliations:** 1 Aix Marseille Univ, Inserm, IRD, SESSTIM, Sciences Economiques & Sociales de la Santé & Traitement de l’Information Médicale, ISSPAM, 13005 Marseille, France; 2 Hôpital St. Joseph, Service d’Hépato-Gastroentérologie, 13008 Marseille, France; 3 Institut National de la Santé et de la Recherche Médicale (INSERM), Institut Pierre Louis d’Epidémiologie et de Santé Publique, Sorbonne Université, 75012 Paris, France; 4 Hôpital Saint-Antoine, Unité de Santé Publique, Assistance Publique-Hôpitaux de Paris (AP-HP), 75012 Paris, France; 5 ANRS|Emerging Infectious Diseases, Department of Clinical Research, 75015 Paris, France; 6 INSERM U1052, CNRS UMR-5286, Cancer Research Center of Lyon (CRCL), 69008 Lyon, France; 7 University of Lyon, Université Claude-Bernard (UCBL), 69007 Lyon, France; 8 Hospices Civils de Lyon (HCL), 69002 Lyon, France; 9 Centre de Recherche des Cordeliers, Sorbonne Université, Inserm, Université Paris Cité, 75019 Paris, France; 10 Hôpital Européen Georges Pompidou, Assistance Publique-Hôpitaux de Paris (AP-HP), 75610 Paris, France; 11 AP-HP Hôpital Paul-Brousse, Centre Hépato-Biliaire, Villejuif, UMR-S 1193, Université Paris-Saclay, FHU HEPATINOV, 94800 Villejuif, France; 12 Université de Paris, Assistance Publique-Hôpitaux de Paris (AP-HP), Hôpital Cochin, Département d’Hépatologie/Addictologie, 75014 Paris, France

**Keywords:** hepatitis C, chronic, cannabis, metabolic syndrome, dyslipidemia, hypertension

## Abstract

Background and Aims. Hepatitis C virus (HCV) infection is associated with the onset of metabolic disorders which constitute risk factors for liver disease progression. Their impact may persist after the HCV infection has been cured. Cannabis use is associated with a lower risk of obesity and diabetes in both general and HCV populations. The associations between cannabis use and both dyslipidemia and hypertension have not yet been studied in persons with chronic HCV infection. Methods. Using cross-sectional data from the French ANRS CO22 Hepather cohort, we used regression models to test for an inverse relationship between cannabis use and (i) dyslipidemia, (ii) hypertension, and (iii) the total number of metabolic disorders. Results. Among the 6364 participants in the study population, both former and current cannabis use were associated with a lower risk of hypertension and fewer metabolic disorders. These results were independent of central obesity. Cannabis use was not associated with dyslipidemia. Conclusions. In people chronically infected with HCV, cannabis use was associated with a lower risk of hypertension and a lower number of metabolic disorders. Post-HCV cure studies are needed to confirm these findings using longitudinal data and to test whether they translate into reduced mortality in this population.

## 1. Introduction

Chronic hepatitis C virus (HCV) infection can lead to a spectrum of liver disease manifestations including cirrhosis and hepatocellular carcinoma. In addition, it is positively associated with metabolic syndrome (MetS), insulin resistance, hepatic steatosis, and the risk of type 2 diabetes and atherosclerosis [1,2]. The incidence of some of these comorbidities is higher in certain populations of people with chronic infection, depending on the HCV genotype [3]. MetS is characterized as the accumulation of at least three of the following five cardiovascular risk factors: central obesity, elevated triglycerides, reduced high-density lipoprotein cholesterol, elevated blood pressure, and elevated fasting glucose [4]. In addition to increasing the risk of cardiovascular diseases, which is generally high in HCV-infected people [5,6], MetS may be a factor in impaired quality of life [5]. The presence of metabolic disorders can also influence the HCV chronicity and virulence, leading to liver disease progression [7,8,9]. Moreover, hepatic steatosis—a risk factor for accelerated fibrogenesis and development of hepatocellular carcinoma [10]—is recognized as the liver manifestation of MetS, and hepatic steatosis and MetS share pathophysiological mechanisms, with insulin resistance playing a pivotal role [11].

Contrary to what was observed with interferon-based therapies, MetS does not seem to affect the sustained virologic response to treatment with direct-acting antivirals (DAA) [12]. HCV cure following DAA treatment generally leads to improvements in glucose homeostasis [12,13,14] but has no long-term impact on glucose metabolism in patients suffering from liver cirrhosis [14,15]. Pre-DAA diabetes increases mortality and liver-related events independently of HCV cure [12], and diabetes is also an independent risk factor of *de novo* hepatocellular carcinoma after DAA treatment [16]. Weight gain is likely to occur after DAA treatment [17], while the impact of overweight and obesity on post-HCV cure cirrhosis and hepatocellular carcinoma development remain unclear [12,18]. There is no hard evidence for the benefit of DAA-based HCV cure on elevated blood pressure [19], and the long-term impact of DAA therapy on serum lipid needs to be investigated [20,21]. All the preceding points highlight the need to prevent and manage metabolic disorders in HCV-infected people, both before and after HCV cure.

We previously showed that cannabis use is inversely associated with diabetes and obesity in HCV-infected people [22,23]. However, no data are available on the associations between cannabis use and both hypertension and dyslipidemia for this population. In the general population, cannabis use tends to be associated with a lower risk of meeting the criteria for metabolic syndrome diagnosis [24,25], which may be mediated by a body weight-lowering effect of cannabis [25]. However, contrasting results have been observed for hypertension [26,27,28,29] and for dyslipidemia [30] when taken individually. These various points, and the fact that cannabis use is a modifiable factor, underline the need to explore the relationships between cannabis use and metabolic disorders in HCV-infected patients in order to (i) increase understanding about the mechanisms related to extrahepatic manifestations of HCV infection, and (ii) document the metabolic impacts of cannabis use, with a view to guiding further research that may benefit both HCV-infected and non-infected people.

In the present study, we aimed to test whether cannabis use in chronically HCV-infected patients was associated with hypertension, dyslipidemia, and the overall number of metabolic disorders before DAA initiation, after adjustment for clinical, sociodemographic and behavioral factors.

## 2. Materials and Methods

### 2.1. Participants and Design

ANRS CO22 Hepather is a French national, prospective, multicenter, observational cohort study of patients with HCV and/or hepatitis B virus (HBV) infection (registered with ClinicalTrials.gov, number NCT01953458). Recruitment exclusion criteria were HIV coinfection, receiving HCV treatment, or having stopped HCV treatment for less than three months at enrolment. Thirty-two expert hospital clinical centers across the country are involved in data collection. The cohort has been extensively described elsewhere [31].

During the enrolment visit, sociodemographic, clinical, and biological data were collected. Follow-up included systematic visits once a year and spontaneous reports for particular events (e.g., HCV or HBV treatment initiation). Written informed consent was obtained from each patient before enrolment. The cohort protocol was approved by the ‘Comité de Protection des Personnes (CPP) Ile de France 3′ Ethics Committee (Paris, France) (#2943, 29 November 2011) and the French Regulatory Authority (ANSM) and designed in accordance with French law for biomedical research and the Declaration of Helsinki.

### 2.2. Data Collection

The present study used patients’ enrolment visit data, collected by a participating physician in an electronic case-report form which was based on a structured questionnaire. Specifically, sociodemographic and clinical data, including sex, age, country of birth, time since HCV diagnosis, HCV genotype, tobacco use, current coffee consumption (number of cups per day), cannabis use, current and past alcohol use (number of standard drinks per day), educational level (highest diploma obtained), employment status, average monthly household income, and the number of adults and children in the household.

Anthropometric measurements (body height, weight, and waist circumference) were performed, and urine and blood samples were taken. Data from blood samples included platelet count (platelets/L), alanine aminotransferase (ALT, IU/L), aspartate aminotransferase (AST, IU/L), and gamma glutamyltransferase (GGT, IU/L) levels.

The physician also collected data on medical comorbidities at enrolment, including hypertension, diabetes, hypertriglyceridemia, and hypercholesterolemia.

### 2.3. Study Population

The study population was composed of patients with chronic HCV infection at cohort enrolment (defined as positive HCV-RNA and anti-HCV antibodies). Patients with HBV coinfection were excluded from the study population, as were those with no data for cannabis use or for variables needed to assess metabolic disorders (i.e., waist circumference, dyslipidemia, hypertension, and diabetes).

### 2.4. Outcomes

We selected three outcomes for the present study. The first, dyslipidemia, was defined as the presence of hypertriglyceridemia or hypercholesterolemia, or receiving treatment for these disorders. The second, hypertension, was defined as the presence of hypertension or receiving treatment for it. The third outcome was the overall number of concurrent metabolic disorders among the following four: dyslipidemia, hypertension, diabetes, and central obesity. The latter was defined as a waist circumference ≥94 cm for men (except for men born in Asia, Central America, or South America, for whom the cut-off was set at 90 cm) and ≥80 cm for women [32].

### 2.5. Explanatory Variables

For both cannabis and tobacco use, participants were classified into ‘current’, ‘former’, or ‘never’ categories. A three-category variable was created to test for a potential dose–response relationship between coffee consumption and the outcomes as follows: (i) 0 cups/day, (ii) 1–2 cups/day, and (iii) ≥3 cups/day. The threshold of 3 cups/day was chosen as it has previously been associated with a protective effect of coffee consumption on liver stiffness and mortality in patients likely to develop liver disease [33].

Alcohol consumption was coded into three categories as follows: abstinent with no history of unhealthy alcohol use, (ii) current moderate alcohol use (i.e., non-abstinent and non-unhealthy use), and (iii) current or past unhealthy alcohol use. Unhealthy alcohol use was defined according to the French National Authority for Health’s guidelines [34]: >3 and >2 standard drinks per day for men and women, respectively.

Poverty was defined as a standard of living lower than 1015 euros per month (the 2015 French poverty threshold) [35]. Standard of living was calculated as disposable monthly income by household consumption unit. Educational level was dichotomized into having at least an upper secondary school certificate or not. Employment status was defined as having a job or not.

Liver fibrosis was assessed with the noninvasive FIB-4 index, calculated using AST level, ALT level, age, and platelet count with the following formula: (AST [IU/L] *age [years])/(ALT [IU/L] * platelet count [10^9^/L])^1/2^. Advanced fibrosis was defined as an FIB-4 index > 3.25 [36,37]. HCV genotype was classified as 1, 3, 4, or 2/5/6/7, because genotypes 1 and 4 are associated with insulin resistance [38] and genotype 3 with viral steatosis [7].

Place of birth was coded as a five-category variable (‘France’, ‘Europe and America’, ‘North Africa and Middle East’, ‘Sub-Saharan Africa’, and ‘Asia’) according to expected genetic and/or geographical proximity. Other explanatory variables used in the analyses were sex, age, and time since HCV diagnosis.

### 2.6. Statistical Analyses

Study population characteristics were compared between never, former, and current cannabis users. Similarly, cannabis use and the presence of metabolic disorders were compared between the study population and patients who were excluded because of missing data. Kruskal–Wallis and Chi-square tests were used for continuous and categorical variables, respectively.

Two separate logistic regression models were used to test for associations between cannabis use and the first two outcomes (i.e., dyslipidemia and hypertension). A negative binomial regression model was used to test for the association between cannabis use and the third outcome (i.e., number of metabolic disorders). For the two logistic models, associations were assessed using (adjusted) odds ratios [(a)OR], while incidence risk ratios were used for the negative binomial model. Only variables with a liberal *p*-value < 0.20 (Wald test) in the univariable analyses were considered eligible for the multivariable models. A backward stepwise selection procedure was used to build the final multivariable models. The Wald test (*p*-value < 0.05) was used to define the variables to maintain in the final models.

As the potential effect of cannabis use on metabolic disorders may be indirect (i.e., due to a lowered corpulence), a sensitivity analysis which adjusted for waist circumference was performed for each outcome. For the two first outcomes, two separate regressions were run including waist circumference as an explanatory variable (as a continuous variable and as a binary variable with the threshold for central obesity). For the third outcome, a regression was run in which central obesity was removed from the list of four disorders considered for the outcome (this number then varied from 0 to three), and waist circumference was included as an explanatory variable (as a continuous variable and as a binary variable with the threshold for central obesity). Finally, as described in a previous article [22], the association between cannabis use and diabetes was assessed using a logistic model similar to that used for dyslipidemia and hypertension.

Statistical analyses were performed using Stata software version 17.0 for Windows (StataCorp LP, College Station, TX, USA).

## 3. Results

### 3.1. Study Population Characteristics

**Figure 1:**
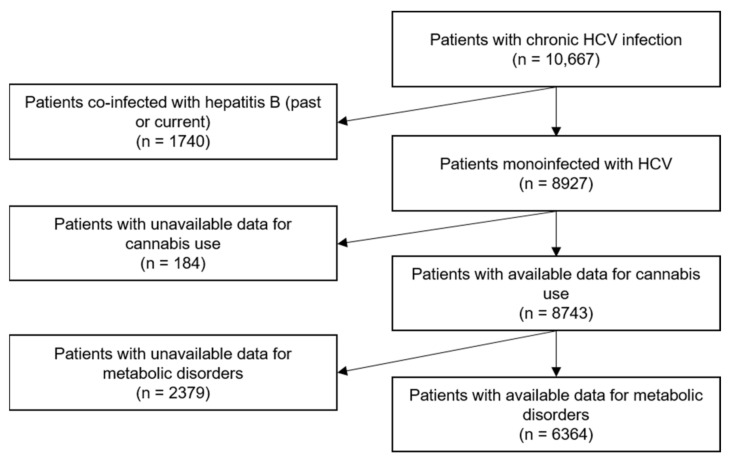
Flow chart of the study population (ANRS CO22 Hepather cohort).

The study population was comprised of 6364 participants (Figure 1). Their characteristics are described globally and according to their cannabis use in Table 1. Over half were men (53.8%), and the median age was 56 years (interquartile range [50–64]). One in eight (12.3 %) were currently using cannabis, and 20.4% were former users. Two thirds (66.3%) had at least one metabolic disorder, the most prevalent being central obesity (Supplementary Table S1).

Participants excluded because of missing data differed significantly but not substantially from those included in terms of place of birth, tobacco use, alcohol use, living in poverty, educational level, employment status, and HCV genotype (data not shown).

### 3.2. Factors Associated with Dyslipidemia

Results from the univariable and multivariable logistic regressions for dyslipidemia are provided in Table 2. Former and current cannabis use were both inversely associated with dyslipidemia only in the univariable analysis. In the multivariable model, older age, moderate alcohol use (vs. abstinent with no history of unhealthy use) and having no job were associated with dyslipidemia, while HCV genotype 3 (vs. genotype 1) was inversely associated. The two sensitivity analyses confirmed the absence of any significant association between cannabis use and the outcome.

### 3.3. Factors Associated with Hypertension

Results from the univariable and multivariable logistic regressions for hypertension are provided in Table 2. Former and current cannabis use were both inversely associated with hypertension in both analyses (adjusted odds ratio [aOR] [95% confidence interval (CI)]: 0.74 [0.62;0.89], *p* = 0.001, and 0.45 [0.36; 0.59], *p* < 10^−3^, respectively). In the multivariable model, being a male, older age, being born in ‘Europe or America’ or in ‘Sub-Saharan Africa’ (vs. in France), not having an upper secondary school certificate, and not having a job were also associated with hypertension.

The two sensitivity analyses confirmed the associations between former and current cannabis use and this outcome (aOR 0.78 and 0.54, respectively, with adjustment for central obesity, and 0.80 and 0.58, respectively, with adjustment for waist circumference as a continuous variable; data not shown).

Results from the univariable and multivariable logistic regressions for diabetes are given in Supplementary Table S2.

### 3.4. Factors Associated with the Number of Metabolic Disorders

Results from the univariable and multivariable negative binomial regressions for the number of metabolic disorders are provided in Table 3. Former and current cannabis use were both inversely associated with this outcome in both analyses (adjusted incidence risk ratio [aIRR] [95% CI]: 0.80 [0.71;0.90], *p* < 10^−3^, and 0.53 [0.44;0.63], *p* < 10^−3^, respectively).

The two sensitivity analyses confirmed the associations between former and current cannabis use and the outcome (aIRR: 0.84 and 0.60, respectively, with adjustment for central obesity, and 0.84 and 0.61, respectively, with adjustment for waist circumference as a continuous variable; data not shown).

## 4. Discussion

In a population of 6364 patients with chronic HCV-infection, both former and current cannabis use were associated with a lower risk of hypertension and a lower overall number of metabolic disorders (among central obesity, dyslipidemia, hypertension, and diabetes). However, cannabis use was not associated with dyslipidemia. These results complement previous findings on the protective effect of cannabis use on obesity and diabetes in the same cohort [22,23]. They also document the effects of cannabis on people with current, untreated, chronic HCV infection.

The inverse relationship we found between cannabis use and hypertension in HCV-infected people adds to the current body of research in different populations. Indeed, existing results on this issue have been very contrasting. For example, in the United States’ National Health and Nutrition Examination Survey (NHNES), Vidot et al. found that current cannabis use in the general population was longitudinally associated with hypertension, a result driven by heavy users [26]. Elsewhere in the U.S., while one cohort found that greater cannabis use in younger adults was significantly associated with lower systolic and diastolic blood pressure [25], another found it was significantly associated with higher systolic blood pressure [39]. However, neither of these opposing associations was significant after multiple adjustment (especially for body mass index and alcohol use). In the NHNES, Alshaarawy et al. found a modest association between recent cannabis use and higher systolic blood pressure (but not diastolic blood pressure or hypertension) [27], as well as greater blood pressure variability in cannabis users [40]. Conversely, a case report highlighted potential benefits of cannabis use on blood pressure stability in the context of autonomic dysreflexia [41]. Recently, Haleem et al. found no association between cannabis use and hypertension incidence in the United States’ National Epidemiologic Survey on Alcohol and Related Conditions [28]. Elsewhere, after three months of cannabis-based treatment, blood pressure declined in 26 older adults with hypertension [29]. There are also conflicting results about the consequences of abrupt cessation of heavy cannabis use on blood pressure; some authors suggest it may cause clinically significant increases in blood pressure in a subset of users [42], while others did not find this result [43].

These many contrasting results in the literature may stem from a multitude of confounding factors such as tobacco use, age (cannabis use is generally more prevalent in younger people, while hypertension becomes more prevalent with older age), stress, and body weight. Moreover, differences in cannabis composition (such as concentrations of cannabidiol and Δ9-tetrahydrocannabinol [44,45]), administration route, and lifetime cumulated exposure—factors which are not easy to collect—may lead to different consequences on blood pressure. Finally, the reliability of blood pressure assessment may suffer from single measurement.

In terms of dyslipidemia, we did not find any association between cannabis use and this outcome. Indeed, literature on the effects of cannabis on lipoproteins is inconsistent [30].

We found a negative relationship between HCV genotype 3 (vs. genotype 1) and dyslipidemia, in line with previous findings for cholesterol in HIV–HCV co-infected people [46]. This effect may be linked to lower peroxisome proliferator-activated receptors γ and α expression [47,48], polymorphism of IL28B gene [49], and/or interference with the late cholesterol synthesis pathway in genotype 3 [50], and more broadly with wide alterations in gene regulatory networks associated with lipid metabolism between genotypes 1 and 3 [51].

In our study population, cannabis use was inversely associated with the overall number of metabolic disorders (among central obesity, dyslipidemia, hypertension, and diabetes). Although our criteria did not exactly match those generally accepted for defining MetS, our findings suggest that cannabis use may reduce the risk of cardiovascular disease in HCV-infected people. In the general population, the number of MetS components was associated with a higher risk of cardiovascular and all-cause mortality [52] and cardiovascular disease incidence [53]. The presence of metabolic disorders is also believed to influence the chronicity and virulence of HCV, leading to liver disease progression [7,8,9]. Moreover, the inverse relationship between cannabis use and the number of metabolic disorders in our study population is likely to translate into a lower risk of MetS for cannabis users.

The sensitivity analyses we performed suggest that the effects of current or former cannabis use on hypertension, and the number of metabolic disorders in this HCV-infected population, were independent of the well-documented effect of cannabis lowering body mass index [25,54,55]. In a previous paper, we highlighted this effect on the HCV population [23]. This finding is very important, as almost half of the participants in our study had no central obesity, a proportion quite similar to that found for body mass index-based obesity in a Taiwanese study where MetS prevalence was higher in lean HCV-infected people than their lean hepatitis-free counterparts [2].

HCV-infected people are at a higher risk of developing insulin resistance through mechanisms independent of body weight [1]. Insulin resistance fosters the development of hepatic steatosis, hepatic fibrosis, and hepatocellular carcinoma. While DAA-based HCV cure improves glycemic control, patients with advanced fibrosis may continue to have a degree of insulin resistance [1]. Therefore, all current means to manage metabolic disorders that may foster insulin resistance and hepatic steatosis in this population, including in people without obesity, are of clinical interest. Moreover, in descriptive analyses, we found that cannabis users were less likely to have advanced liver fibrosis than non-users (Table 1). A direct or indirect effect of cannabis use on liver fibrosis or liver disease has been previously suggested [33,56], but such an hypothesis would deserve further studies [57]. An indirect protective effect of cannabis use on liver fibrosis (through the prevention of metabolic disorders), as suggested by our results (advanced liver fibrosis was associated with the number of metabolic disorders), would also call for further studies in order to be explored. The fact that older age constituted a risk factor for all three study outcomes highlights the need for proper clinical follow-up of ageing HCV-infected people in order to comprehensively prevent and/or manage metabolic disorders and their associated complications. Furthermore, as chronic inflammation, immune activation, and hepatic fibrosis do not necessarily regress after HCV cure [58,59], managing other risk factors for cardiovascular diseases and hepatocellular carcinoma is crucial. In line with other studies, having a job was inversely associated with all three study outcomes, revealing an association between lower socioeconomic status and MetS [60,61,62]. Social determinants should therefore not be neglected when considering the global health of the HCV-infected population.

Our results are independent of any possible interference by DAA treatment, as data were measured before treatment initiation. Chronic inflammation caused by HCV infection is particularly high before HCV cure. It is not completely resolved following HCV cure [63,64]. As cannabis and cannabinoids possess anti-inflammatory properties [65], and given that chronic inflammation is closely related to the onset of metabolic disorders [66], we hypothesize that cannabis use lowers the risk of metabolic disorders partly through a decrease in chronic inflammation. Our results highlight the need to replicate the analyses presented here in post-HCV cure contexts. Our results suggest that it may be relevant to take exposure to cannabinoids into account when assessing the risk profile of HCV patients in terms of metabolic disorders. While we cannot recommend cannabis use, the benefits we found may justify guiding users toward low-risk use [67] rather than ignoring their cannabis use behavior. Moreover, more cannabinoid-based research is needed in order to understand the mechanisms involved in metabolic regulation by cannabinoids and to develop safe therapeutics.

Our study has several strengths. First, its sample size is large. Second, we collected demographic, clinical, and socio-behavioral variables. Although data for some well-known risk factors for metabolic disorders were not collected (e.g., diet quality and physical activity), we may expect that adjustment for socio-demographic variables—which are generally associated with these risk factors [68,69,70]—at least partially captured their effects.

In terms of limitations, cannabis use was self-reported, opening the door to potential desirability bias. However, any such bias would have been equally likely in people with and without metabolic disorders. Furthermore, we did not have data to match our definition of MetS with the generally agreed upon definition, and therefore any conclusion made concerning the metabolic disorders we used and MetS and its components should be carefully considered. Further prospective studies with longitudinal collection of data and additional confounding factors are needed in order to confirm our results. Finally, the exclusive enrolment within hospital services may have biased our study population.

## 5. Conclusions

In a large cohort of people with chronic HCV infection living in France, current or former cannabis use was associated with a lower risk of hypertension and a lower number of metabolic disorders. However, no association was found for dyslipidemia. Post-HCV cure studies are needed to confirm these findings using longitudinal data. Future research should also explore the biological mechanisms underlying these potential benefits of cannabis use, and test whether they translate into reduced mortality in this population.

## Figures and Tables

**Table 1:** Study population characteristics, according to cannabis use (ANRS CO22 Hepather cohort, *n* = 6364 patients with chronic HCV infection).

Variables (% Missing Values)	Whole Study Population (*n* = 6364)	Cannabis Use			
		Never (*n* = 4283)	Former (*n* = 1296)	Current (*n* = 785)	*p*-Value †
	*n* (%) or Median [IQR]	*n* (%)	*n* (%)	*n* (%)	
**Sex** (0)					
Male	3422 (53.8)	1877 (43.8)	927 (71.5)	618 (78.7)	<10^−3^
Female	2942 (46.2)	2406 (56.2)	369 (28.5)	167 (21.3)	
**Age (years)** (0)					
Median [IQR]	56 [50;64]	59 [51;68]	52 [49;56]	50 [46;55]	<10^−3^
**Place of birth** (0)					
France	4684 (73.6)	2924 (68.3)	1092 (84.3)	668 (85.1)	<10^−3^
Europe and America ‡	478 (7.5)	340 (7.9)	95 (7.3)	43 (5.5)	
North Africa and Middle East	576 (9.1)	458 (10.7)	71 (5.5)	47 (6.0)	
Sub-Saharan Africa §	423 (6.6)	392 (9.2)	19 (1.5)	12 (1.5)	
Asia	200 (3.1)	166 (3.9)	19 (1.5)	15 (1.9)	
**Coffee consumption** (0.7)					
0 cups/day	1801 (28.5)	1375 (32.3)	273 (21.2)	153 (19.6)	<10^−3^
1–2 cups/day	2537 (40.1)	1781 (41.9)	488 (37.9)	268 (34.4)	
≥3 cups/day	1981 (31.3)	1096 (25.8)	526 (40.9)	359 (46.0)	
**Tobacco use** (0)					
Never	2399 (37.7)	2318 (54.1)	55 (4.2)	26 (3.3)	<10^−3^
Former	1750 (27.5)	1131 (26.4)	547 (42.2)	72 (9.2)	
Current	2214 (34.8)	833 (19.5)	694 (53.5)	687 (87.5)	
**Alcohol consumption** ¶ (0.4)					
Abstinent with no history of unhealthy use	2728 (43.0)	2302 (53.9)	274 (21.3)	152 (19.6)	<10^−3^
Moderate use	2529 (39.9)	1552 (36.3)	614 (47.7)	363 (46.7)	
Current or past unhealthy use	1080 (17.0)	418 (9.8)	400 (31.1)	262 (33.7)	
**Living in poverty** † † (2.6)					
No	4317 (69.6)	3015 (72.4)	881 (69.6)	421 (54.7)	<10^−3^
Yes	1885 (30.4)	1151 (27.6)	385 (30.4)	349 (45.3)	
**Educational level** (1.1)					
<upper secondary school certificate	3400 (54.0)	2281 (53.8)	645 (50.4)	474 (60.8)	<10^−3^
≥ upper secondary school certificate	2895 (46.0)	1955 (46.2)	635 (49.6)	305 (39.2)	
**Employment status** (0.4)					
Having no job	3526 (55.6)	2648 (62.1)	506 (39.1)	372 (47.6)	<10^−3^
Having a job	2815 (44.4)	1617 (37.9)	788 (60.9)	410 (52.4)	
**Advanced liver fibrosis** ‡ ‡ (6.5)					
No	4404 (74.0)	2897 (72.1)	913 (76.3)	594 (80.7)	<10^−3^
Yes	1545 (26.0)	1120 (27.9)	283 (23.7)	142 (19.3)	
**Time since HCV diagnosis (years)** (2.0)					
Median [IQR]	14.0 [6.8;19.8]	13.7 [6.8;19.7]	14.3 [6.3;19.7]	15.4 [7.8;20.5]	0.005
**HCV genotype**					
1	4133 (65.9)	2850 (67.6)	804 (63.0)	479 (61.6)	<10^−3^
3	780 (12.4)	351 (8.3)	256 (20.1)	173 (22.3)	
4	761 (12.1)	485 (11.5)	174 (13.6)	102 (13.1)	
2/5/6/7	594 (9.5)	529 (12.6)	42 (3.3)	23 (3.0)	
**Central obesity** (0)					
No	2841 (44.6)	1639 (38.3)	682 (52.6)	520 (66.2)	<10^−3^
Yes	3523 (55.4)	2644 (61.7)	614 (47.4)	265 (33.8)	
**Dyslipidemia** (0)					
No	5844 (91.8)	3886 (90.7)	1217 (93.9)	741 (94.4)	<10^−3^
Yes	520 (8.2)	397 (9.3)	79 (6.1)	44 (5.6)	
**Hypertension** (0)					
No	4532 (71.2)	2769 (64.7)	1066 (82.3)	697 (88.8)	<10^−3^
Yes	1832 (28.8)	1514 (35.3)	230 (17.7)	88 (11.2)	
Diabetes (0)					
No	5547 (87.2)	3631 (84.8)	1176 (90.7)	740 (94.3)	<10^−3^
Yes	817 (12.8)	652 (15.2)	120 (9.3)	45 (5.7)	
**Number of metabolic disorders** § § (0)					
0	2145 (33.7)	1136 (26.5)	569 (43.9)	440 (56.1)	<10^−3^
1	2376 (37.3)	1628 (38.0)	483 (37.3)	265 (33.8)	
2	1304 (20.5)	1054 (24.6)	185 (14.3)	65 (8.3)	
3	448 (7.0)	389 (9.1)	46 (3.5)	13 (1.7)	
4	91 (1.4)	76 (1.8)	13 (1.0)	2 (0.3)	

† Chi-square and Kruskall-Wallis tests were used for categorical and continuous variables, respectively. ‡ The category ‘Europe and America’ included participants from South America (*n* = 19), North America (*n* = 15), Central America (*n* = 4), Australia (*n* = 2), and Russia (*n* = 32). § The category ‘Sub-Saharan Africa’ included participants from the Caribbean (*n* = 10). ¶ Unhealthy alcohol use was defined as >3 and >2 standard drinks per day for men and women, respectively, in accordance with the French National Authority for Health’s guidelines [34]. † † Poverty was defined as a standard of living lower than the 2015 French poverty threshold (1015 euros) [35]. ‡ ‡ Advanced liver fibrosis was defined as a FIB-4 score > 3.25 [36]. § § Among central obesity, dyslipidemia, hypertension, and diabetes. HCV, hepatitis C virus; IQR, interquartile range.

**Table 2:** Factors associated with dyslipidemia and hypertension (univariable and multivariable logistic regression models, ANRS CO22 Hepather cohort, *n* = 6364).

	Univariable Analyses				Multivariable Analysis			
Variables	Dyslipidemia (*n* = 6364)	*p*-Value	Hypertension (*n* = 6364)	*p*-Value	Dyslipidemia (*n* = 6219)	*p*-Value	Hypertension (*n* = 6248)	*p*-Value
	OR [95% CI]		OR [95% CI]		aOR [95% CI]		aOR [95% CI]	
**Sex**								
Male (ref.)	1		1				1	
Female	1.01 [0.84;1.20]	0.955	1.44 [1.29;1.61]	<10^−3^			0.87 [0.77;0.99]	0.034
**Age (years)**	1.04 [1.03;1.04]	<10^−3^	1.08 [1.07;1.09]	<10^−3^	1.03 [1.02;1.04]	<10^−3^	1.07 [1.06;1.08]	<10^−3^
**Place of birth**		0.428		<10^−3^				<10^−3^
France (ref.)	1		1				1	
Europe and America †	0.85 [0.59;1.21]	0.362	1.26 [1.03;1.55]	0.024			1.33 [1.07;1.67]	0.011
North Africa and Middle East	0.86 [0.62;1.20]	0.381	1.34 [1.11;1.61]	0.002			1.04 [0.85;1.28]	0.695
Sub-Saharan Africa ‡	0.73 [0.49;1.09]	0.126	2.25 [1.84;2.76]	<10^−3^			2.74 [2.19;3.43]	<10^−3^
Asia	0.81 [0.46;1.40]	0.445	0.86 [0.62;1.20]	0.389			1.06 [0.73;1.54]	0.754
**Coffee consumption**		0.067		<10^−3^				0.034
0 cups/day (ref.)	1		1				1	
1–2 cups/day	1.28 [1.02;1.60]	0.032	1.00 [0.88;1.13]	0.959			1.05 [0.90;1.21]	0.542
≥3 cups/day	1.07 [0.84;1.36]	0.600	0.53 [0.46;0.62]	<10^−3^			0.86 [0.73;1.01]	0.070
**Cannabis use**		<10^−3^		<10^−3^				<10^−3^
Never (ref.)	1		1				1	
Former	0.64 [0.49;0.82]	<10^−3^	0.39 [0.34;0.46]	<10^−3^			0.74 [0.62;0.89]	0.001
Current	0.58 [0.42;0.80]	0.001	0.23 [0.18;0.29]	<10^−3^			0.45 [0.36;0.59]	<10^−3^
**Tobacco use**		0.026		<10^−3^				
Never (ref.)	1		1					
Former	1.04 [0.84;1.29]	0.723	0.72 [0.63;0.82]	<10^−3^				
Current	0.78 [0.63;0.97]	0.023	0.32 [0.28;0.37]	<10^−3^				
**Alcohol consumption** §		0.100		<10^−3^		0.006		
Abstinent with no history of unhealthy use (ref.)	1		1		1			
Moderate use	1.19 [0.98;1.44]	0.084	0.65 [0.57;0.73]	<10^−3^	1.39 [1.14;1.70]	0.001		
Current or past unhealthy use	0.93 [0.71;1.22]	0.591	0.62 [0.53;0.73]	<10^−3^	1.15 [0.87;1.52]	0.331		
**Living in poverty** ¶								
No (ref.)	1		1					
Yes	0.77 [0.63;0.95]	0.014	0.96 [0.85;1.08]	0.517				
**Educational level**								
<upper secondary school certificate (ref.)	1		1				1	
≥upper secondary school certificate	0.98 [0.82;1.17]	0.832	0.67 [0.60;0.75]	<10^−3^			0.79 [0.70;0.90]	<10^−3^
**Employment status**								
Having no job (ref.)	1		1		1		1	
Having a job	0.58 [0.48;0.70]	<10^−3^	0.38 [0.34;0.43]	<10^−3^	0.80 [0.64;1.00]	0.046	0.80 [0.69;0.92]	0.002
**Advanced liver fibrosis** † †								
No (ref.)	1		1					
Yes	0.98 [0.79;1.21]	0.843	1.66 [1.47;1.88]	<10^−3^				
**Time since HCV diagnosis (years)**	1.01 [1.00;1.02]	0.026	1.01 [1.00;1.02]	0.003				
**HCV genotype**		<10^−3^		<10^−3^		0.005		
1 (ref.)	1		1		1			
3	0.51 [0.36;0.73]	<10^−3^	0.61 [0.51;0.74]	<10^−3^	0.63 [0.44;0.91]	0.013		
4	1.00 [0.76;1.32]	0.993	0.95 [0.80;1.13]	0.563	1.22 [0.92;1.62]	0.176		
2/5/6/7	1.48 [1.13;1.94]	0.005	1.36 [1.14;1.63]	0.001	1.31 [0.99;1.73]	0.059		

† The category ‘Europe and America’ included participants from South America (*n* = 19), North America (*n* = 15), Central America (*n* = 4), Australia (*n* = 2), and Russia (*n* = 32). ‡ The category ‘Sub-Saharan Africa’ included participants from the Caribbean (*n* = 10). § Unhealthy alcohol use was defined as >2 and >3 standard drinks per day for women and men, respectively, in accordance with the French National Authority for Health’s guidelines [34] ¶ Poverty was defined as a standard of living lower than the 2015 French poverty threshold (1015 euros) [35]. † † Advanced liver fibrosis was defined as a FIB-4 score > 3.25 [36]. aOR, adjusted odds ratio; CI, confidence interval; HCV, hepatitis C virus; OR, odds ratio. Cells are empty for variables not kept in the final multivariable models (multivariable analyses columns).

**Table 3:** Factors associated with the number of metabolic disorders among central obesity, dyslipidemia, hypertension, and diabetes (univariable and multivariable negative binomial regression models, ANRS CO22 Hepather cohort, *n* = 6364).

Variables	Univariable Analyses (*n* = 6364)	*p*-Value	Multivariable Analysis (*n* = 5779)	*p*-Value
	IRR [95% CI]		aIRR [95% CI]	
**Sex**				
Male (ref.)	1		1	
Female	1.09 [1.02;1.17]	0.017	0.82 [0.76;0.88]	<10^−3^
**Age (years)**	1.04 [1.04;1.05]	<10^−3^	1.03 [1.03;1.04]	<10^−3^
**Place of birth**		<10^−3^		<10^−3^
France (ref.)	1		1	
Europe and America †	1.07 [0.93;1.22]	0.358	1.08 [0.94;1.22]	0.277
North Africa and Middle East	1.38 [1.24;1.54]	<10^−3^	1.10 [0.98;1.23]	0.104
Sub-Saharan Africa ‡	1.57 [1.39;1.76]	<10^−3^	1.53 [1.35;1.74]	<10^−3^
Asia	0.95 [0.75;1.20]	0.675	1.08 [0.85;1.37]	0.547
**Coffee consumption**		<10^−3^		0.011
0 cups/day (ref.)	1		1	
1–2 cups/day	1.06 [0.97;1.15]	0.187	1.11 [1.02;1.21]	0.011
≥3 cups/day	0.71 [0.64;0.78]	<10^−3^	1.00 [0.90;1.11]	0.962
**Cannabis use**		<10^−3^		<10^−3^
Never (ref.)	1		1	
Former	0.55 [0.50;0.62]	<10^−3^	0.80 [0.71;0.90]	<10^−3^
Current	0.38 [0.32;0.44]	<10^−3^	0.53 [0.44;0.63]	<10^−3^
**Tobacco use**		<10^−3^		
Never (ref.)	1			
Former	0.90 [0.83;0.98]	0.011		
Current	0.54 [0.49;0.59]	<10^−3^		
**Alcohol consumption** §		<10^−3^		
Abstinent with no history of unhealthy use (ref.)	1			
Moderate use	0.79 [0.73;0.85]	<10^−3^		
Current or past unhealthy use	0.81 [0.73;0.90]	<10^−3^		
**Living in poverty** ¶				
No (ref.)	1			
Yes	1.02 [0.94;1.10]	0.695		
**Educational level**				
<upper secondary school certificate (ref.)	1		1	
≥upper secondary school certificate	0.76 [0.71;0.82]	<10^−3^	0.87 [0.81;0.94]	<10^−3^
**Employment status**				
Having no job (ref.)	1		1	
Having a job	0.51 [0.47;0.55]	<10^−3^	0.80 [0.73;0.88]	<10^−3^
**Advanced liver fibrosis** † †				
No (ref.)	1		1	
Yes	1.47 [1.37;1.59]	<10^−3^	1.13 [1.05;1.22]	0.002
**Time since HCV diagnosis (years)**	1.01 [1.00;1.01]	0.001		
**HCV genotype**		<10^−3^		0.039
1 (ref.)	1		1	
3	0.68 [0.60;0.78]	<10^−3^	0.94 [0.83;1.08]	0.404
4	1.03 [0.92;1.16]	0.553	1.16 [1.03;1.31]	0.013
2/5/6/7	1.21 [1.09;1.35]	0.001	0.97 [0.87;1.08]	0.528

† The category ‘Europe and America’ included participants from South America (*n* = 19), North America (*n* = 15), Central America (*n* = 4), Australia (*n* = 2), and Russia (*n* = 32). ‡ The category ‘Sub-Saharan Africa’ included participants from the Caribbean (*n* = 10). § Unhealthy alcohol use was defined as >2 and >3 standard drinks per day for women and men, respectively, in accordance with the French National Authority for Health’s guidelines [34]. ¶ Poverty was defined as a standard of living lower than the 2015 French poverty threshold (1015 euros) [35]. † † Advanced liver fibrosis was defined as a FIB-4 score > 3.25 [36]. aIRR, adjusted incidence risk ratio; CI, confidence interval; HCV, hepatitis C virus; IRR, incidence risk ratio. Cells are empty for variables not kept in the final multivariable models (multivariable analysis columns).

## Data Availability

Not applicable.

## Supplementary Materials

The following supporting information can be downloaded at: https://www.mdpi.com/article/10.3390/jcm11206135/s1, Table S1: Presence of metabolic disorders in the study population at enrolment (ANRS CO22 Hepather cohort, *n* = 6364); Table S2: Factors associated with diabetes (univariable and multivariable logistic regression models, ANRS CO22 Hepather cohort, *n* = 6364).

## Scientific Committee

- *Voting members*:

Marc Bourlière (Hôpital St Joseph, Marseille), Jérôme Boursier (CHU Angers, Angers, France), Fabrice Carrat (Scientific Coordinator, Hôpital Saint-Antoine, Paris, France), Patrizia Carrieri (INSERM U912, Marseille, France), Elisabeth Delarocque-Astagneau (Inserm UMR1181, Paris), Victor De Ledinghen (Hôpital Haut-Lévêque, Pessac, Bordeaux, France), Céline Dorival (UPMC & INSERM U1136, Paris, France), Hélène Fontaine (Hôpital Cochin, Paris, France), Slim Fourati (Hôpital Henri Mondor, Créteil, France), Chantal Housset (Inserm UMR-S938 1 IFR65, Paris), Dominique Larrey (Hôpital Saint Eloi, Montpellier, France), Pierre Nahon (Hôpital Jean Verdier, Bondy, France), Georges-Philippe Pageaux (Hôpital Saint Eloi, Montpellier, France), Ventzislava Petrov-Sanchez (ANRS, Paris, France), Stanislas Pol (Principal Investigator, Hôpital Cochin, Paris, France), Mathias Bruyand (Agence Nationale de Santé Publique, Saint Maurice, France), Linda Wittkop (ISPED-INSERM U897, Bordeaux, France), Fabien Zoulim (Hospices Civils de Lyon, Lyon, France), Jessica Zucman-Rossi (Inserm U674/1162, Paris).

- *Non-voting members*:

Marianne L’hennaff (ARCAT-TRT-5-CHV, France), Michèle Sizorn (SOS hépatites, France); one representative from the INSERM-ANRS-MIE Pharmacovigilance team, Paris, France (Anaïs Boston, Alpha Diallo), Carole Cagnot (INSERM-ANRS-MIE, Paris, France), one member from Inserm Transfert, Paris, France (Alice Bousselet, Mireille Caralp), and one representative from each pharmaceutical company (MSD, Gilead, Abbvie).
