# Supplementary material for: Cannabis Use Is Inversely Associated with Metabolic Disorders in Hepatitis C-Infected Patients (ANRS CO22 Hepather Cohort)
Source: J Clin Med. 2022 Oct 18;11(20):6135. doi: 10.3390/jcm11206135 (PMC9605108; doi:10.3390/jcm11206135)
Supplement: Supplementary file 1 [file jcm-11-06135-s001.zip › SuppTable1_JCM_revis.pdf]

Journal of
Clinical Medicine

MDPI

Supplementary Table S1. Presence of metabolic disorders in the study population at enrolment (ANRS CO22 Hepather cohort, n=6364).

	Participants with central obesity (n=3523)		Participants with dyslipidemia (n=520)		Participants with hypertension (n=1832)		Participants with diabetes (n=817)	
	n	%	n	%	n	%	n	%
Participants with one metabolic disorder (n=2376)	1823	76.7	101	4.3	343	14.4	109	4.6
Participants with two metabolic disorders (n=1304)	1186	91.0	156	12.0	976	74.8	290	22.2
Participants with three metabolic disorders (n=448)	423	94.4	172	38.4	422	94.2	327	73.0
Participants with all four metabolic disorders (n=91)	91	100	91	100	91	100	91	100
