# Supplementary material for: Cannabis Use Is Inversely Associated with Metabolic Disorders in Hepatitis C-Infected Patients (ANRS CO22 Hepather Cohort)
Source: J Clin Med. 2022 Oct 18;11(20):6135. doi: 10.3390/jcm11206135 (PMC9605108; doi:10.3390/jcm11206135)
Supplement: Supplementary file 1 [file jcm-11-06135-s001.zip › SuppTable2_JCM_revis.pdf]

Journal of
Clinical Medicine

MDPI

Supplementary Table S2: Factors associated with diabetes (univariable and multivariable logistic regression models, ANRS CO22 Hepather cohort, n=6364)

Variables	Univariable analyses Diabetes (n=6364) OR [95% CI]	P-value	Multivariable analyses Diabetes (n=5845) aOR [95% CI]	P-value
**Gender**				
Male (ref.)	1		1	
Female	0.75 [0.64 – 0.87]	< 10^{-3}	0.58 [0.49 – 0.69]	< 10^{-3}
**Age (years)**	1.04 [1.03 – 1.05]	< 10^{-3}	1.03 [1.02 – 1.03]	< 10^{-3}
**Place of birth**				
France (ref.)	1	**< 10^{-3}**	1	**< 10^{-3}**
Europe and America †	0.95 [0.70 – 1.29]	0.721	0.95 [0.68 – 1.31]	0.741
North Africa and Middle East	2.50 [2.02 – 3.10]	< 10^{-3}	2.02 [1.60 – 2.56]	< 10^{-3}
Sub-Saharan Africa ‡	2.16 [1.68 – 2.77]	< 10^{-3}	2.73 [2.03 – 3.69]	< 10^{-3}
Asia	1.21 [0.79 – 1.85]	0.377	1.37 [0.87 – 2.15]	0.172
**Coffee consumption**				
0 cup/day (ref.)	1	**< 10^{-3}**	1	**0.044**
1-2 cups/day	1.11 [0.93 – 1.32]	0.238	1.25 [1.03 – 1.53]	0.024
≥ 3 cups/day	0.68 [0.55 – 0.83]	< 10^{-3}	1.05 [0.83 – 1.34]	0.671
**Cannabis use**				
Never (ref.)	1	**< 10^{-3}**	1	**< 10^{-3}**
Former	0.57 [0.46 – 0.70]	< 10^{-3}	0.75 [0.59 – 0.96]	0.024
Current	0.34 [0.25 – 0.46]	< 10^{-3}	0.37 [0.25 – 0.55]	< 10^{-3}
**Tobacco use**				
Never (ref.)	1	**< 10^{-3}**	1	**0.017**
Former	1.08 [0.91 – 1.29]	0.362	1.35 [1.10 – 1.65]	0.004
Current	0.61 [0.51 – 0.74]	< 10^{-3}	1.17 [0.91 – 1.50]	0.224
**Alcohol consumption §**				
Abstinent without history of unhealthy use (ref.)	1	**< 10^{-3}**		
Moderate use	0.67 [0.57 – 0.79]	< 10^{-3}		
Current or past unhealthy use	0.96 [0.79 – 1.18]	0.717		
**Living in poverty ¶**				
No (ref.)	1			
Yes	1.35 [1.15 – 1.57]	< 10^{-3}		
**Educational level**				
< upper secondary school certificate (ref.)	1		1	
≥ upper secondary school certificate	0.63 [0.54 – 0.73]	< 10^{-3}	0.70 [0.59 – 0.82]	< 10^{-3}
**Employment status**				

Having no job (ref.)	1		1	
Having a job	0.44 [0.38 – 0.52]	$< 10^{-3}$	0.68 [0.56 – 0.83]	$< 10^{-3}$
**Advanced liver fibrosis † †**				
No (ref.)	1		1	
Yes	2.30 [1.97 – 2.69]	$< 10^{-3}$	1.80 [1.51 – 2.14]	$< 10^{-3}$
**Time since HCV diagnosis (years)**	1.01 [1.00 – 1.02]	0.144		
**HCV genotype**				
1 (ref.)	1	**0.007**		
3	0.73 [0.57 – 0.95]	0.017		
4	1.26 [1.02 – 1.56]	0.036		
2/5/6/7	1.04 [0.81 – 1.34]	0.746		

† The category 'Europe and America' included participants from South America (n=19), North America (n=15), Central America (n=4), Australia (n=2) and Russia (n=32). ‡ The category 'Sub-Saharan Africa' included participants from the Caribbean (n=10). § Unhealthy alcohol use was defined as > 2 and > 3 standard drinks per day for women and men, respectively, in accordance with the French National Authority for Health [34] ¶ Poverty was defined as a standard of living lower than the 2015 French poverty threshold (1015 euros) [35] † † Advanced liver fibrosis was defined as a FIB-4 score > 3.25 [36]. aOR, adjusted odds ratio; CI, confidence interval; HCV, hepatitis C virus; OR, odds ratio.
